# Methyl 3-[(*tert*-but­oxy­carbon­yl)amino]­benzoate

**DOI:** 10.1107/S2414314625005425

**Published:** 2025-06-24

**Authors:** Murugesan Ponmagaram, Krishnan Saranraj, Karuppiah Muruga Poopathi Raja

**Affiliations:** a Chemical Biology and Biophysical Laboratory Department of Physical Chemistry School of Chemistry Madurai Kamaraj University,Madurai - 625 021 Tamilnadu India; b Chemical Biology and Biophysical Laboratory Department of Chemistry School of Physical Sciences Sabarmati Building Tejaswini Hills Central University of Kerala, Periye Kasaragod District - 671 320 Kerala India; University of Aberdeen, United Kingdom

**Keywords:** crystal structure, aromatic γ-amino acid, *meta*-amino benzoic acid

## Abstract

In the extended structure of the title compound, C_13_H_17_NO_4_, mol­ecular pairs are connected *via* N—H⋯O and C—H⋯O hydrogen bonds, generating inversion dimers characterized by *R*^2^_2_(10) graph-set motifs. These dimers further associate through N—H⋯O and C—H⋯O inter­actions, forming supra­molecular layers lying parallel to the (104) crystallographic plane. Aromatic π–π stacking inter­actions and C—H⋯π contacts contribute to the tri-periodic supra­molecular architecture.

## Abstract 2

In the extended structure of the title compound, mol­ecular pairs are connected *via* N—H⋯O and C—H⋯O hydrogen bonds, generating inversion dimers characterized by *R*^2^_2_(10) graph-set motifs. These dimers further associate through N—H⋯O and C—H⋯O inter­actions, forming supra­molecular layers lying parallel to the (104) crystallographic plane. Aromatic π–π stacking inter­actions and C—H⋯π contacts contribute to the tri-periodic supra­molecular architecture.

## Graphical abstract

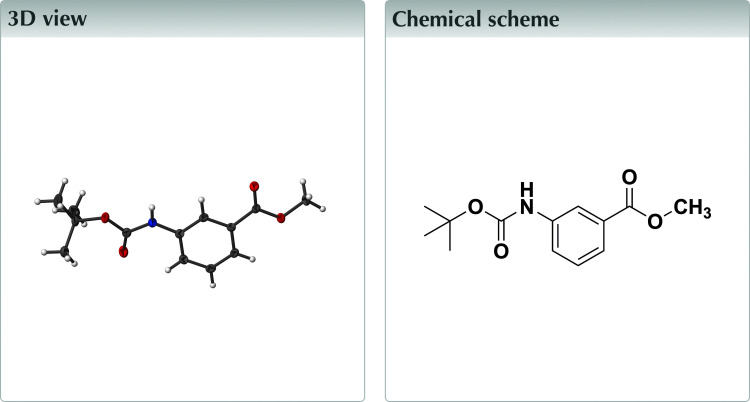

## Structure description

**Figure 1:**
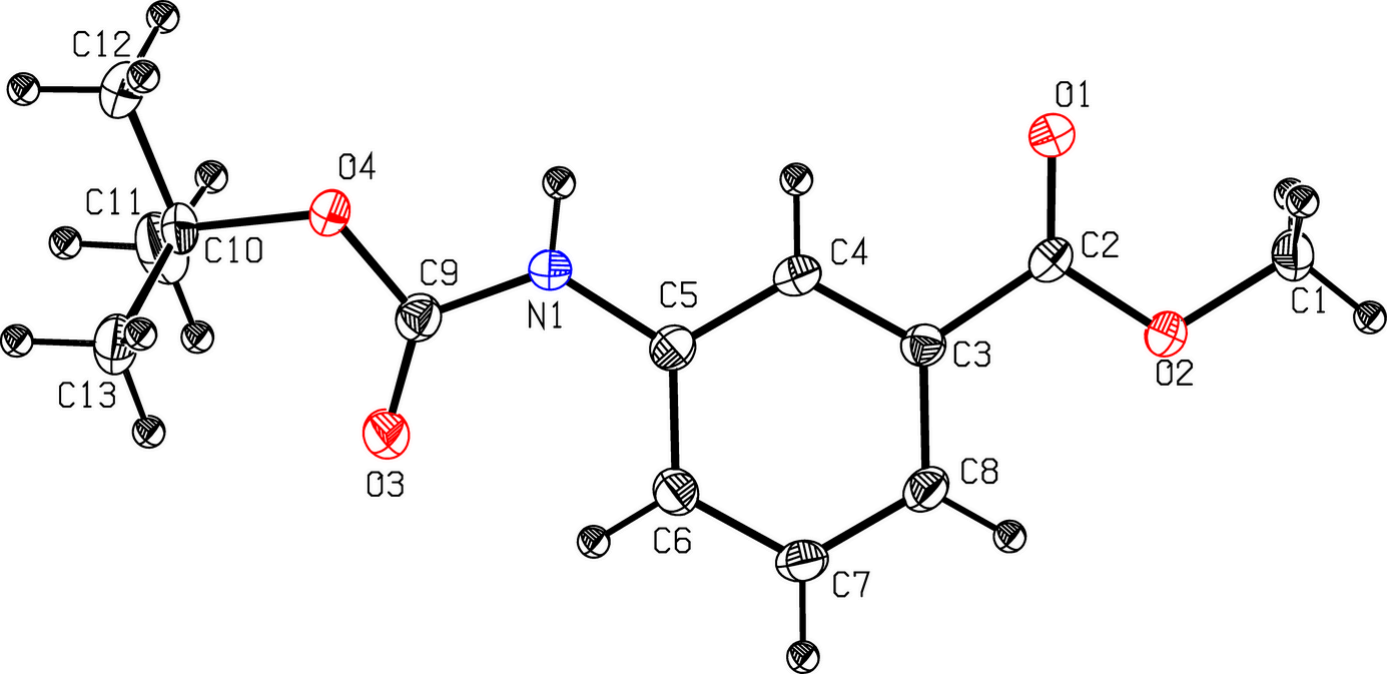
The mol­ecular structure of the title compound with displacement ellipsoids drawn at the 30% probability level.

*meta*-Amino­benzoic acid has uses in organic synthesis, chemical biology, and materials science (Benke *et al.*, 2020 ▸). It facilitates the formation of supra­molecular sheets, rendering it a valuable component for the design of peptide-based frameworks and amyloid-mimetic fibrillar architectures (Boruah & Roy, 2022 ▸). Its electron-rich aromatic framework and amino-substituted functionality promote the synthesis of a wide range of bioactive heterocycles, making it a valuable precursor in medicinal chemistry, agrochemical design and functional material development (Kundu *et al.*, 2002 ▸; Maity *et al.*, 2013 ▸; Dutta *et al.*, 2023 ▸). As part of our studies in this area, we now describe the synthesis and structure of the title compound (Fig. 1 ▸).

The linking angle (C4—C5—N1—C9) between the aromatic ring (C3–C8) and the amide group (N1—C9=O3) is 170.99 (17)°, indicating near coplanarity. The amide–carbamate conformation, defined by atoms C5—N1—C9—O4 = 174.66 (16)°, indicates an extended *transoid* conformation. This conformation appears to facilitate optimal inter­molecular N—H⋯O hydrogen bonding. The torsion angle between the amide group and the Boc moiety (N1—C9—O4—C10) is 170.57 (14)°, with one of the C atoms of the *tert*-butyl group almost in the same plane as the amide group, one below and one above. Such behaviour is consistent with other Boc-protected aromatic amides reported in the Cambridge Structural Database (CSD; Groom *et al.*, 2016 ▸), where Boc groups often adopt staggered conformations relative to the adjacent peptide or aryl systems to reduce unfavourable steric inter­actions. The ester group, defined by atoms O1—C2—C3—C8, exhibits a torsion angle of −173.73 (18)°, indicating an *anti* conformation.

**Figure 2:**
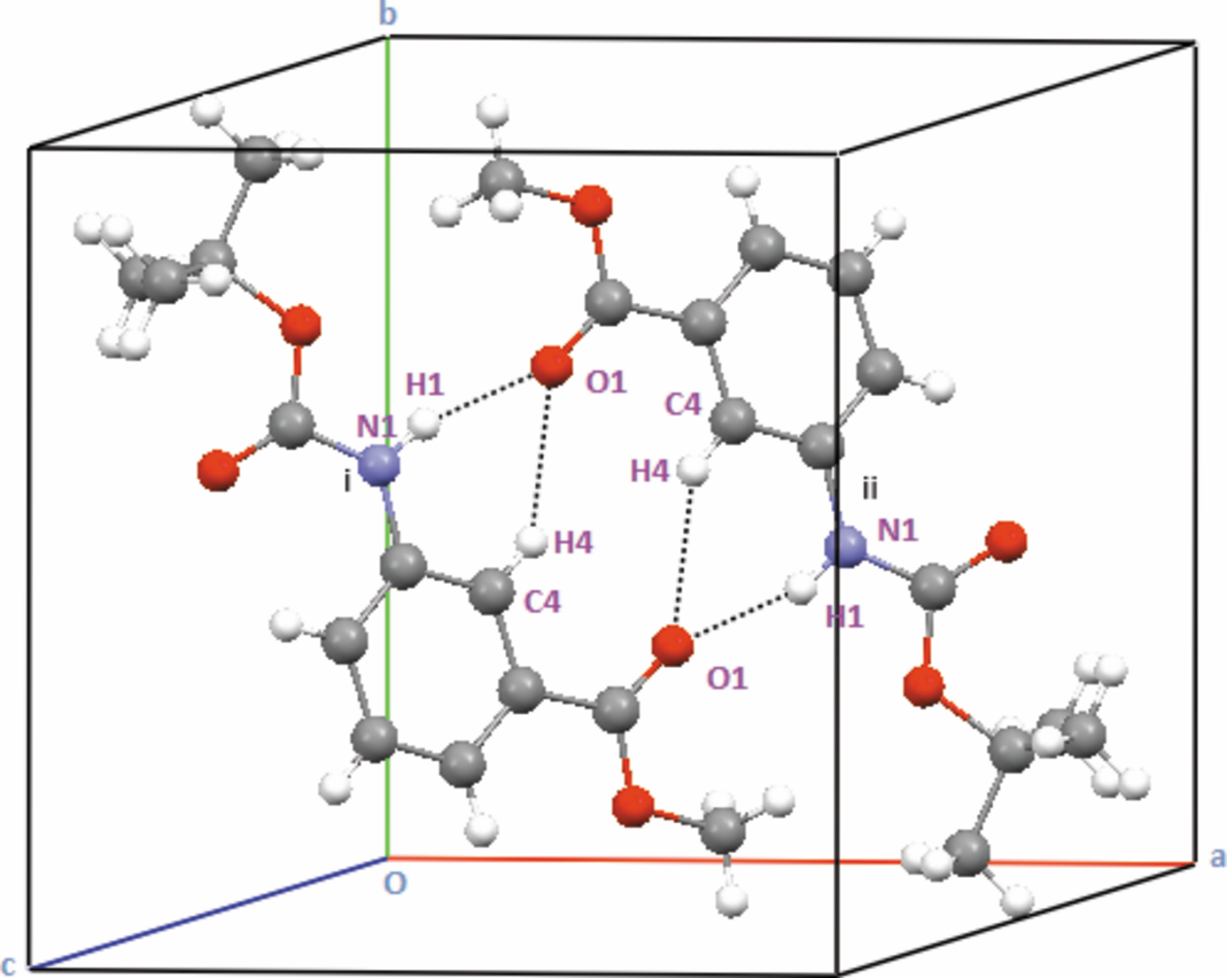
Partial packing of the title compound showing C—H⋯O and N—H⋯O hydrogen-bonded inversion dimers with 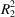
(10), 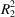
(12) and 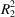
(14) graph-set motifs. The two independent mol­ecules are labelled as i and ii. [Symmetry codes: (i) 1 − *x*, 1 − *y*, 1 − *z*; (ii) 1 − *x*, 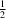 + *y*, 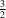 − *z*.]

**Figure 3:**
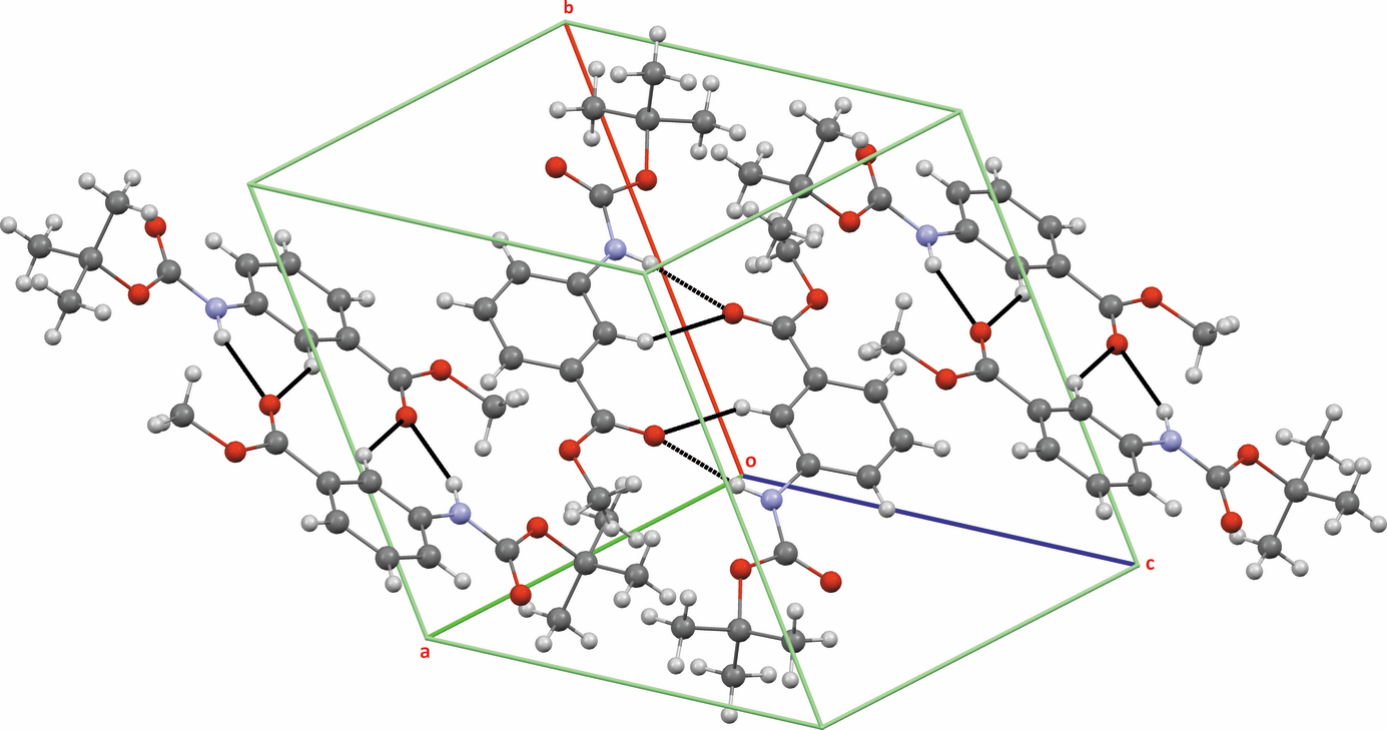
The crystal packing viewed approximately along [111] with the N—H⋯O and C—H⋯O hydrogen bonds shown as dashed lines.

In the extended structure, the mol­ecules are assembled into inversion dimers (Table 1 ▸, Fig. 2 ▸) through pairwise N—H⋯O and C—H⋯O hydrogen bonds, forming 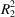
(10), 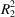
(12) and 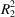
(14) ring motifs that generate zigzag ribbons propagating along the *c*-axis direction (Fig. 3 ▸). An N—H⋯O hydrogen bond is observed between the carbamoyl and carboxyl­ate groups; additionally the ribbons are inter­connected by C—H⋯O hydrogen bonds, resulting in a double-chain architecture (Fig. 3 ▸). The twisting and non-coplanarity among the fragments appear to be a compromise between steric demands (particularly from the Boc group) and the desire for favourable inter­molecular inter­actions such as hydrogen bonds and stacking; additionally the ribbons are inter­connected by C—H⋯O hydrogen bonds, resulting in a double-chain architecture (Fig. 3 ▸).

## Synthesis and crystallization

10 mmol (1.368 g) of *meta*-amino benzoic acid were dissolved in 10 ml of a 5% *w*/*v* sodium carbonate solution in a round-bottom flask. Subsequently, 12 mmol (2.619 g) of Boc-anhydride in 10 ml of dry tetra­hydro­furan (THF) were added. The resulting mixture, characterized by a pH of 12, was subjected to stirring for a duration of 12 h. The THF solvent was evaporated utilizing a rotavapor, and the resulting solution was adjusted to a pH of 2 using 2 *N* HCl. Upon three extractions with ethyl acetate, the organic layer underwent drying with anhydrous sodium sulfate and subsequent evaporation, resulting in a yield of 3.85 g (91%). In an ice bath, a combination of 20 ml of anhydrous methanol and 6 ml of thionyl chloride was prepared, followed by the addition of 20 ml (1.50 g) of the Boc-protected amino acid. The sealed flask was left to stir overnigh. Methanol was then removed through distillation and diethyl ether was introduced, yielding 1.32 g (89%) of the title compound. The purification process encompassed the utilization of silica gel along with a mixture of ethyl acetate and petroleum ether. The final products appeared as a white, colourless powder. Crystallization was accomplished by the gradual evaporation of mixed ethanol–water solvents, leading to the formation of stable, colourless crystals.

## Refinement

Crystal data, data collection and structure refinement details are summarized in Table 2 ▸.

## Supplementary Material

Crystal structure: contains datablock(s) global, I. DOI: 10.1107/S2414314625005425/hb4522sup1.cif

Structure factors: contains datablock(s) I. DOI: 10.1107/S2414314625005425/hb4522Isup2.hkl

Supporting information file. DOI: 10.1107/S2414314625005425/hb4522Isup3.cml

CCDC reference: 2360910

Additional supporting information:  crystallographic information; 3D view; checkCIF report

## Figures and Tables

**Table 1:** Hydrogen-bond geometry (Å, °)

*D*—H⋯*A*	*D*—H	H⋯*A*	*D*⋯*A*	*D*—H⋯*A*
N1—H1⋯O1^i^	0.86	2.17	2.987 (2)	160
C4—H4⋯O1^i^	0.93	2.41	3.211 (2)	145
C13—H13*C*⋯O1^ii^	0.96	2.55	3.496 (3)	167

Symmetry codes: (i) 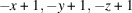; (ii) 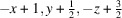.

**Table 2:** Experimental details

Crystal data	
Chemical formula	C_13_H_17_NO_4_
*M*_r_	251.28
Crystal system, space group	Monoclinic, *P*2_1_/*c*
Temperature (K)	296
*a*, *b*, *c* (Å)	10.944 (3), 11.234 (3), 11.377 (3)
β (°)	112.481 (4)
*V* (Å^3^)	1292.5 (5)
*Z*	4
Radiation type	Mo *K*α
μ (mm^−1^)	0.10
Crystal size (mm)	0.21 × 0.19 × 0.18

Data collection	
Diffractometer	Bruker *SMART* APEXII CCD
Absorption correction	Multi-scan (*SADABS*; Krause *et al.*, 2015 ▸)
*T*_min_, *T*_max_	0.631, 0.746
No. of measured, independent and observed [*I* > 2σ(*I*)] reflections	17731, 3220, 1966
*R*_int_	0.075
(sin θ/λ)_max_ (Å^−1^)	0.668

Refinement	
*R*[*F*^2^ > 2σ(*F*^2^)], *wR*(*F*^2^), *S*	0.050, 0.121, 1.01
No. of reflections	3220
No. of parameters	168
H-atom treatment	H-atom parameters constrained
Δρ_max_, Δρ_min_ (e Å^−3^)	0.25, −0.24

Computer programs: *APEX2* and *SAINT*(Bruker, 2008 ▸), *SHELXS97* (Sheldrick, 2008 ▸), *SHELXL2019/2* (Sheldrick, 2015 ▸), *ORTEP-3 for Windows* (Farrugia, 2012 ▸) and *publCIF* (Westrip, 2010 ▸).

## full crystallographic data

### Methyl 3-[(tert-butoxycarbonyl)amino]benzoate Crystal data

**Table:**

C_13_H_17_NO_4_	*F*(000) = 752
*M**_r_* = 251.28	*D*_x_ = 1.291 Mg m^−^^3^
Monoclinic, *P*2_1_/*c*	Mo *K*α radiation, λ = 0.71073 Å
*a* = 10.944 (3) Å	Cell parameters from 1967 reflections
*b* = 11.234 (3) Å	θ = 2.0–28.4°
*c* = 11.377 (3) Å	µ = 0.10 mm^−^^1^
β = 112.481 (4)°	*T* = 296 K
*V* = 1292.5 (5) Å^3^	Block, colourless
*Z* = 4	0.21 × 0.19 × 0.18 mm

### Methyl 3-[(tert-butoxycarbonyl)amino]benzoate Data collection

**Table:**

Bruker SMART APEXII CCD diffractometer	1966 reflections with *I* > 2σ(*I*)
Radiation source: fine-focus sealed tube	*R*_int_ = 0.075
ω and φ scans	θ_max_ = 28.4°, θ_min_ = 2.0°
Absorption correction: multi-scan (SADABS; Krause *et al.*, 2015)	*h* = −14→12
*T*_min_ = 0.631, *T*_max_ = 0.746	*k* = −14→14
17731 measured reflections	*l* = −15→15
3220 independent reflections	

### Methyl 3-[(tert-butoxycarbonyl)amino]benzoate Refinement

**Table:**

Refinement on *F*^2^	Hydrogen site location: inferred from neighbouring sites
Least-squares matrix: full	H-atom parameters constrained
*R*[*F*^2^ > 2σ(*F*^2^)] = 0.050	*w* = 1/[σ^2^(*F*_o_^2^) + (0.0491*P*)^2^ + 0.2412*P*] where *P* = (*F*_o_^2^ + 2*F*_c_^2^)/3
*wR*(*F*^2^) = 0.121	(Δ/σ)_max_ < 0.001
*S* = 1.01	Δρ_max_ = 0.25 e Å^−^^3^
3220 reflections	Δρ_min_ = −0.23 e Å^−^^3^
168 parameters	Extinction correction: SHELXL-2019/2 (Sheldrick 2008), Fc^*^=kFc[1+0.001xFc^2^λ^3^/sin(2θ)]^-1/4^
0 restraints	Extinction coefficient: 0.0096 (15)
Primary atom site location: structure-invariant direct methods	

### Methyl 3-[(tert-butoxycarbonyl)amino]benzoate Special details

**Table:**

Geometry. All esds (except the esd in the dihedral angle between two l.s. planes) are estimated using the full covariance matrix. The cell esds are taken into account individually in the estimation of esds in distances, angles and torsion angles; correlations between esds in cell parameters are only used when they are defined by crystal symmetry. An approximate (isotropic) treatment of cell esds is used for estimating esds involving l.s. planes.
Refinement. N and C-bound H atoms were positioned geometrically (C–H = 0.93–0.98 Å) and allowed to ride on their parent atoms, with U_iso_(H) = 1.5U_eq_(C) for methyl H atoms and 1.2U_eq_(C) for all other H atoms.

### Methyl 3-[(tert-butoxycarbonyl)amino]benzoate Fractional atomic coordinates and isotropic or equivalent isotropic displacement parameters (Å^2^)

**Table:**

	*x*	*y*	*z*	*U*_iso_*/*U*_eq_	
C1	0.62827 (18)	0.10342 (16)	0.47993 (18)	0.0243 (4)	
H1A	0.583451	0.121193	0.391158	0.036*	
H1B	0.643567	0.019253	0.490723	0.036*	
H1C	0.711362	0.144702	0.512517	0.036*	
C2	0.52600 (17)	0.25857 (15)	0.54767 (17)	0.0186 (4)	
C3	0.43796 (17)	0.29156 (15)	0.61424 (16)	0.0183 (4)	
C4	0.39765 (17)	0.40983 (16)	0.60627 (17)	0.0199 (4)	
H4	0.426532	0.463943	0.560604	0.024*	
C5	0.31466 (17)	0.44819 (15)	0.66576 (16)	0.0196 (4)	
C6	0.27334 (18)	0.36633 (16)	0.73476 (17)	0.0216 (4)	
H6	0.218801	0.390662	0.775977	0.026*	
C7	0.31357 (18)	0.24816 (16)	0.74209 (17)	0.0230 (4)	
H7	0.284882	0.194009	0.787865	0.028*	
C8	0.39548 (18)	0.20972 (16)	0.68254 (17)	0.0212 (4)	
H8	0.421773	0.130509	0.687994	0.025*	
C9	0.18725 (18)	0.62189 (16)	0.69033 (17)	0.0216 (4)	
C10	0.08789 (18)	0.81923 (16)	0.68615 (18)	0.0239 (4)	
C11	−0.0445 (2)	0.7864 (2)	0.58521 (19)	0.0373 (6)	
H11A	−0.037471	0.783865	0.503700	0.056*	
H11B	−0.109167	0.844840	0.583632	0.056*	
H11C	−0.071121	0.709729	0.604166	0.056*	
C12	0.1331 (2)	0.94171 (18)	0.6618 (2)	0.0420 (6)	
H12A	0.221113	0.956535	0.722260	0.063*	
H12B	0.074302	1.001277	0.670662	0.063*	
H12C	0.132422	0.944532	0.577242	0.063*	
C13	0.0882 (2)	0.81423 (17)	0.81919 (18)	0.0284 (5)	
H13A	0.055317	0.738297	0.832519	0.043*	
H13B	0.032576	0.876232	0.829252	0.043*	
H13C	0.176745	0.824988	0.880178	0.043*	
N1	0.27956 (14)	0.56926 (13)	0.65412 (14)	0.0209 (4)	
H1	0.320586	0.614865	0.620673	0.025*	
O1	0.57322 (13)	0.32966 (11)	0.49628 (12)	0.0249 (3)	
O2	0.54760 (12)	0.14127 (10)	0.54830 (12)	0.0227 (3)	
O3	0.11464 (13)	0.57148 (11)	0.73180 (13)	0.0292 (3)	
O4	0.19151 (12)	0.74073 (11)	0.67341 (12)	0.0243 (3)	

### Methyl 3-[(tert-butoxycarbonyl)amino]benzoate Atomic displacement parameters (Å^2^)

**Table:**

	*U*^11^	*U*^22^	*U*^33^	*U*^12^	*U*^13^	*U*^23^
C1	0.0260 (10)	0.0196 (10)	0.0315 (11)	0.0011 (8)	0.0157 (9)	−0.0032 (8)
C2	0.0199 (9)	0.0145 (9)	0.0196 (10)	−0.0001 (7)	0.0056 (8)	0.0002 (7)
C3	0.0208 (9)	0.0168 (9)	0.0176 (9)	−0.0020 (7)	0.0075 (8)	−0.0011 (7)
C4	0.0223 (9)	0.0167 (9)	0.0208 (10)	−0.0012 (7)	0.0085 (8)	0.0023 (7)
C5	0.0198 (9)	0.0179 (9)	0.0201 (10)	−0.0015 (7)	0.0067 (8)	−0.0010 (7)
C6	0.0214 (10)	0.0216 (10)	0.0237 (10)	−0.0010 (8)	0.0109 (8)	−0.0019 (8)
C7	0.0285 (10)	0.0193 (10)	0.0228 (10)	−0.0032 (8)	0.0114 (8)	0.0021 (8)
C8	0.0259 (10)	0.0143 (9)	0.0229 (10)	−0.0004 (8)	0.0091 (8)	0.0000 (8)
C9	0.0237 (10)	0.0189 (9)	0.0219 (10)	0.0002 (8)	0.0085 (8)	−0.0011 (8)
C10	0.0251 (10)	0.0221 (10)	0.0294 (11)	0.0088 (8)	0.0157 (9)	0.0024 (8)
C11	0.0312 (12)	0.0526 (15)	0.0273 (12)	0.0144 (10)	0.0102 (10)	−0.0005 (10)
C12	0.0519 (14)	0.0235 (11)	0.0660 (16)	0.0143 (10)	0.0397 (13)	0.0107 (11)
C13	0.0308 (11)	0.0268 (11)	0.0271 (11)	0.0097 (9)	0.0107 (9)	−0.0003 (9)
N1	0.0232 (8)	0.0166 (8)	0.0279 (9)	0.0004 (6)	0.0153 (7)	0.0021 (6)
O1	0.0309 (8)	0.0182 (7)	0.0320 (8)	0.0013 (6)	0.0193 (6)	0.0026 (6)
O2	0.0301 (7)	0.0150 (7)	0.0279 (7)	−0.0001 (5)	0.0166 (6)	−0.0018 (5)
O3	0.0321 (8)	0.0212 (7)	0.0433 (9)	−0.0006 (6)	0.0246 (7)	−0.0010 (6)
O4	0.0274 (7)	0.0163 (7)	0.0349 (8)	0.0040 (5)	0.0183 (6)	0.0027 (6)

### Methyl 3-[(tert-butoxycarbonyl)amino]benzoate Geometric parameters (Å, º)

**Table:**

C1—O2	1.446 (2)	C9—O3	1.209 (2)
C1—H1A	0.9600	C9—O4	1.352 (2)
C1—H1B	0.9600	C9—N1	1.363 (2)
C1—H1C	0.9600	C10—O4	1.487 (2)
C2—O1	1.216 (2)	C10—C11	1.511 (3)
C2—O2	1.338 (2)	C10—C13	1.514 (3)
C2—C3	1.482 (2)	C10—C12	1.523 (3)
C3—C4	1.392 (2)	C11—H11A	0.9600
C3—C8	1.393 (2)	C11—H11B	0.9600
C4—C5	1.392 (2)	C11—H11C	0.9600
C4—H4	0.9300	C12—H12A	0.9600
C5—C6	1.392 (2)	C12—H12B	0.9600
C5—N1	1.406 (2)	C12—H12C	0.9600
C6—C7	1.391 (3)	C13—H13A	0.9600
C6—H6	0.9300	C13—H13B	0.9600
C7—C8	1.383 (2)	C13—H13C	0.9600
C7—H7	0.9300	N1—H1	0.8600
C8—H8	0.9300		
			
O2—C1—H1A	109.5	O4—C10—C11	109.09 (15)
O2—C1—H1B	109.5	O4—C10—C13	111.44 (15)
H1A—C1—H1B	109.5	C11—C10—C13	112.67 (17)
O2—C1—H1C	109.5	O4—C10—C12	101.85 (14)
H1A—C1—H1C	109.5	C11—C10—C12	111.49 (17)
H1B—C1—H1C	109.5	C13—C10—C12	109.80 (17)
O1—C2—O2	122.88 (16)	C10—C11—H11A	109.5
O1—C2—C3	124.08 (16)	C10—C11—H11B	109.5
O2—C2—C3	113.04 (15)	H11A—C11—H11B	109.5
C4—C3—C8	120.08 (16)	C10—C11—H11C	109.5
C4—C3—C2	117.20 (16)	H11A—C11—H11C	109.5
C8—C3—C2	122.72 (16)	H11B—C11—H11C	109.5
C3—C4—C5	120.87 (16)	C10—C12—H12A	109.5
C3—C4—H4	119.6	C10—C12—H12B	109.5
C5—C4—H4	119.6	H12A—C12—H12B	109.5
C6—C5—C4	118.86 (17)	C10—C12—H12C	109.5
C6—C5—N1	123.80 (16)	H12A—C12—H12C	109.5
C4—C5—N1	117.34 (16)	H12B—C12—H12C	109.5
C7—C6—C5	120.04 (17)	C10—C13—H13A	109.5
C7—C6—H6	120.0	C10—C13—H13B	109.5
C5—C6—H6	120.0	H13A—C13—H13B	109.5
C8—C7—C6	121.20 (17)	C10—C13—H13C	109.5
C8—C7—H7	119.4	H13A—C13—H13C	109.5
C6—C7—H7	119.4	H13B—C13—H13C	109.5
C7—C8—C3	118.95 (17)	C9—N1—C5	126.77 (15)
C7—C8—H8	120.5	C9—N1—H1	116.6
C3—C8—H8	120.5	C5—N1—H1	116.6
O3—C9—O4	125.57 (16)	C2—O2—C1	115.44 (14)
O3—C9—N1	126.05 (17)	C9—O4—C10	120.18 (13)
O4—C9—N1	108.38 (15)		
			
O1—C2—C3—C4	6.4 (3)	C2—C3—C8—C7	179.86 (16)
O2—C2—C3—C4	−172.91 (15)	O3—C9—N1—C5	−4.7 (3)
O1—C2—C3—C8	−173.73 (18)	O4—C9—N1—C5	174.66 (16)
O2—C2—C3—C8	7.0 (2)	C6—C5—N1—C9	−10.3 (3)
C8—C3—C4—C5	−0.1 (3)	C4—C5—N1—C9	170.99 (17)
C2—C3—C4—C5	179.82 (16)	O1—C2—O2—C1	−1.8 (2)
C3—C4—C5—C6	0.6 (3)	C3—C2—O2—C1	177.44 (15)
C3—C4—C5—N1	179.40 (16)	O3—C9—O4—C10	−10.1 (3)
C4—C5—C6—C7	−0.8 (3)	N1—C9—O4—C10	170.57 (14)
N1—C5—C6—C7	−179.51 (17)	C11—C10—O4—C9	−62.9 (2)
C5—C6—C7—C8	0.5 (3)	C13—C10—O4—C9	62.1 (2)
C6—C7—C8—C3	0.0 (3)	C12—C10—O4—C9	179.17 (16)
C4—C3—C8—C7	−0.2 (3)		

### Methyl 3-[(tert-butoxycarbonyl)amino]benzoate Hydrogen-bond geometry (Å, º)

**Table:**

*D*—H···*A*	*D*—H	H···*A*	*D*···*A*	*D*—H···*A*
N1—H1···O1^i^	0.86	2.17	2.987 (2)	160
C4—H4···O1^i^	0.93	2.41	3.211 (2)	145
C13—H13*C*···O1^ii^	0.96	2.55	3.496 (3)	167

Symmetry codes: (i) −*x*+1, −*y*+1, −*z*+1; (ii) −*x*+1, *y*+1/2, −*z*+3/2.
